# Mobility Modification Alleviates Environmental Influence on Incident Mobility Difficulty among Community-Dwelling Older People: A Two-Year Follow-Up Study

**DOI:** 10.1371/journal.pone.0154396

**Published:** 2016-04-22

**Authors:** Merja Rantakokko, Erja Portegijs, Anne Viljanen, Susanne Iwarsson, Taina Rantanen

**Affiliations:** 1 Gerontology Research Center and Department of Health Sciences, University of Jyvaskyla, Jyvaskyla, Finland; 2 Department of Health Sciences, Lund University, Lund, Sweden; Institute of Nutrition, GERMANY

## Abstract

**Background:**

Environmental barriers increase risk for mobility difficulties in old age. Mobility difficulty is preceded by a phase where people try to postpone a difficulty through mobility modification. We studied whether perceived environmental mobility barriers outdoors correlate with mobility modification and mobility difficulty, predict development of mobility difficulty over a two-year follow-up, and whether mobility modification alleviates the risk for difficulty.

**Methods:**

At baseline, 848 people aged 75–90 were interviewed face-to-face. Telephone follow-up interviews were conducted one (n = 816) and two years (n = 761) later. Environmental barriers to mobility were self-reported using a15-item structured questionnaire at baseline, summed and divided into tertiles (0, 1 and 2 or more barriers). Mobility difficulty was assessed as self-reported ability to walk 2 km at all assessment points and categorized into ‘no difficulty’, ‘no difficulty but mobility modifications’ (reducing frequency, stopping walking, using an aid, slowing down or resting during the performance) and ‘difficulty’.

**Results:**

At baseline, 212 participants reported mobility modifications and 356 mobility difficulties. Those reporting one or multiple environmental barriers had twice the odds for mobility modifications and up to five times the odds for mobility difficulty compared to those reporting no environmental barriers. After multiple adjustments for health and functioning, reporting multiple environmental barriers outdoors continued to predict the development of incident mobility difficulty over the two-year follow-up. Mobility modifications attenuated the association.

**Conclusion:**

For older people who successfully modify their performance, environmental influence on incident mobility difficulty can be diminished. Older people use mobility modification to alleviate environmental press on mobility.

## Introduction

Mobility outside home is a prerequisite for independent living in old age. Mobility modifications are compensatory strategies that enable people to maintain their possibility to move outdoors when experiencing functional decline [1]. These include modifying task performance, such as walking more slowly, walking less often or using assistive devices. Successful modification increases the possibilities to maintain participation in society and in valued activities [2].

The concept of preclinical mobility disability, which is characterised by the use of mobility modifications, was introduced by Fried and colleagues [3] who found that some older people report no difficulty in walking, although they have made changes in their way of performing the mobility task. Preclinical mobility disability was seen as a state between high and low functioning [4]. This notion has since been confirmed, and it has been shown that mobility modifications are on the pathway to manifest mobility disability (for a review, see e.g. Higgins et al. 2014 [5]). Previous studies have mainly focused on the association between individual health determinants of mobility modifications [5–7] and strategies for coping with different mobility tasks [8]. There is little information on how environmental mobility barriers outdoors influence mobility modification in walking longer distances.

The environmental docility hypothesis [9, 10] posits that people with reduced competencies are more vulnerable to environmental press and are at a higher risk for maladaptive behaviour, for example, in disability in mobility tasks. Achieving person-environment fit, for example through mobility modification, may attenuate the disability risk associated with environmental press. Environmental factors associated with incident mobility disability [11, 12] can be seen as factors exacerbating the disablement process, while activity accommodations act as “buffers” that are used to avoid or slow down the process [13]. However, whether the disability risks stemming from environmental mobility barriers outdoors can in fact be alleviated through mobility modification has not been studied. One earlier study showed that indoor mobility barriers, such as steps in doorways or living areas, correlated with task modifications in mobility, such as using mobility devices, relying on help from another person or changing mobility behaviour [6]. However, disability as an outcome was not analysed.

The aim was to study whether perceived environmental mobility barriers outdoors are associated with mobility modification and manifest mobility difficulty among community-dwelling older people. In addition, we studied whether environmental mobility barriers predict the development of incident mobility difficulty, assessed as difficulty walking 2 km, and whether mobility modification alleviates this risk over a two-year follow-up.

We hypothesized that environmental mobility barriers outdoors would correlate with mobility modification and mobility difficulty in walking 2 km and predict incident mobility difficulty over the follow-up [11, 12, 14]. We also hypothesized that mobility modification may be used as a means to achieve person-environment fit, that is, a balance between environmental press and individual competence [9], and that mobility modification attenuates the association between perceived environmental mobility barriers and incident mobility difficulty.

## Methods

### Study design and participants

This study was based on cross-sectional analyses of baseline data and prospective annual follow-up data over two-year period gathered in connection with the “Life-space mobility in old age” (LISPE) project. LISPE is a two-year prospective cohort study on the individual and environmental determinants of life-space mobility among community-dwelling older people in central Finland. The study protocol, methods and non-response analyses have been reported in detail previously [15, 16]. Briefly, 848 75- to 90-year-old people were interviewed in their homes during spring 2012. Follow-up telephone interviews were conducted one (n = 816) and two years (n = 761) later. During the two-year period, 41 participants died, 15 were admitted to institutional care, and 12 were not re-interviewed due to a decline in the ability to communicate. Other reasons for attrition were relocation outside the study area (n = 6), poor health (n = 5), not willing to continue (n = 6), and not reached (n = 2) [16].

The LISPE project was approved by the Ethical Committee of the University of Jyväskylä, Finland. Participants were informed about the project and they signed a written informed consent prior to the baseline interview.

#### Mobility modification

Mobility was assessed as perceived difficulty in walking 2 km. The same standardized questionnaire at the baseline face-to-face interview, and telephone interviews at the one-year and two-year follow-ups were used. The participant was asked whether she/he had difficulties in walking 2 km. The response options were: 1) able to manage without difficulty; 2) able to manage with some difficulty; 3) able to manage with great deal of difficulty; 4) able to manage only with the help of another person; and 5) unable to manage even with help. Those who reported being able to manage without difficulty were asked about their use of five different mobility modifications, using a structured questionnaire that has been shown to be a valid instrument for capturing early signs of mobility decline[4]. The question was formulated as follows: “Have you noticed any of the following changes in your ability to walk 2 km?” The yes/no response option concerned using an aid, reduced task frequency or given up doing the task, performing the task more slowly and resting during performance of the task. If the participant reported any of these mobility modifications, they were categorized as using mobility modification. Three mobility categories were then created; 1) no difficulties, 2) no difficulty but mobility modification 3) manifest mobility difficulties (minor, major difficulties or unable to perform the task).

For the longitudinal analyses, mobility modification was dichotomized (any modification vs. none). Those who did not report difficulties at baseline, but reported manifest mobility difficulty at either the one- or two-year follow-up interview, were categorized as having incident manifest mobility difficulty.

#### Perceived environmental barriers to outdoor mobility

Perceived barriers in the outdoor environment were assessed using the “Checklist for perceived environmental barriers to outdoor mobility” (PENBOM), which is a 15-item questionnaire designed to identify environmental barriers (present/absent) that people perceive as hindering their possibilities for outdoor mobility [17]. The perceived environmental mobility barriers included poor street conditions, high curbs, hills in nearby environment, long distance to services, lack of benches, lack of benches in winter, noisy environment, busy traffic, dangerous crossroads, cyclists on walkways, snow and ice, insecurity due to other pedestrians, cars or services vans on walkways, poor lighting, and lack of pedestrian zones. In the analyses, the sum of the environmental barriers identified as present (yes) was calculated and then divided into tertiles (0,1 and 2 or more barriers).

#### Covariates

As covariates, we included variables known to affect mobility difficulty. Age and gender were derived from the national registers. Other information was obtained in the face-to-face interviews at baseline. Years of education was self-reported. Cognitive functioning was assessed with the Mini-Mental State Examination (MMSE) [18]. Participants were asked whether they lived alone or with someone else (a spouse, children, grandchildren, siblings or other relatives). Self-reported chronic conditions were obtained from a list of 22 physician-diagnosed chronic diseases and an open question [15]. After the data collection, a physician checked the relevance of the diseases reported in the answer to the open question. Chronic conditions were then categorized by the physician. For the present analyses, we included chronic conditions known to affect mobility difficulty, namely diabetes and categories of pulmonary, circulatory, cardiac, and neurological diseases.

Physical performance was objectively assessed by the Short Physical Performance Battery (SPPB)[19], which comprises assessments of standing balance, walking speed over 2.44 meters, and timed chair rises (five times). Each task is rated on a scale from 0 to 4 and a sum score calculated (range 0–12) when at least two tests were completed. Higher scores indicate better performance.

#### Statistical analyses

Participant characteristics are described using means and standard deviations (SD) or percentages in accordance with the baseline mobility categories. Differences between groups were tested with the chi square or one-way analysis of variance (ANOVA). Associations between type of mobility modification and number of environmental barriers were tested with the chi square test.

In the cross-sectional setting, multinomial logistic regression analyses were used to study the associations between environmental barriers outdoors and the mobility categories. All the baseline participants (n = 848) were included in these analyses. Nine participants had missing information on physical performance and were excluded from the corresponding analysis. The model was first adjusted for age and gender, after which all the covariates (physical performance, cognitive functioning, living alone, years of education and diabetes and pulmonary, cardiac, circulatory and neurological diseases) were included in the second model.

In the longitudinal setting, logistic regression analyses were conducted to determine the influence of the environmental mobility barriers on the development of incident mobility difficulty over the follow-up period. In order to study naturally occurring changes in mobility, participants with manifest mobility difficulty at baseline were excluded, leaving 492 participants for the analyses. Of these, 33 had missing information on incident mobility difficulty (14 deceased, 4 admitted to institutional care, 4 relocated outside the study area, 11 unable to communicate, or unwilling or unable to participate in the follow-up interviews due to poor health). Data were not imputed. The logistic regression model was first adjusted for age and gender, after which all the covariates (physical performance, cognitive functioning, living alone, years of education and diabetes and pulmonary, cardiac, circulatory and neurological diseases) were included. Finally baseline mobility modifications (dichotomized as any vs. no modifications) were added to the model to study whether it would attenuate the association between environmental barriers and incident mobility difficulty.

Analyses were performed using IBM SPSS version 22.0 (SPSS Inc., Chicago, IL). When p < .05 or the 95% confidence intervals (CIs) did not include 1, the results were regarded as statistically significant.

## Results

### Cross-sectional findings

The mean age of the participants at baseline was 80.6 (SD 4.3), and 62% of them were women. A total of 212 (25%) participants reported mobility modifications and 356 (42%) mobility difficulties at baseline. Table 1 shows participant characteristics according to mobility group. Participants with mobility modification formed an intermediate group between the participants reporting no mobility difficulty and those reporting manifest mobility difficulty.

**Table 1:** Baseline characteristics according to mobility categorizations among community-dwelling people (n = 848).

Characteristics	No difficultiesn = 280	Mobility modificationsn = 212	Mobility difficultiesn = 356	P-value
	% (n)	% (n)	% (n)	
Women	53.9 (151)	58.5 (124)	70.5 (251)	< .001
Living alone	40.0 (112)	53.1 (112)	64.0 (228)	< .001
Number of perceived environmental barriers				< .001
0	49.6 (139)	32.5 (69)	18.8 (67)	
1	20.4 (57)	22.2 (47)	20.5 (73)	
2 ≥	30.0 (84)	45.3 (96)	60.7 (216)	
Diabetes	9.6 (26)	18.4 (39)	23.3 (83)	< .001
Pulmonary disease	17.1 (48)	18.4 (39)	28.9 (103)	.001
Circulatory disease	51.8 (145)	65.6 (139)	76.7 (273)	< .001
Cardiac disease	28.2 (79)	37.7 (80)	55.6 (198)	< .001
Neurological disease	5.0 (14)	5.7 (12)	9.8 (35)	.039
	Mean (SD)	Mean (SD)	Mean (SD)	
Age	78.9 (3.6)	80.3 (4.3)	82.1 (4.1)	< .001
MMSE score	26.6 (2.5)	26.1 (2.8)	25.8 (3.0)	.003
Education in years	10.3 (4.5)	9.9 (3.7)	8.8 (4.0)	< .001
SPPB score	10.8 (1.4)	10.1 (1.9)	8.4 (3.0)	< .001

SPPB, Short Physical Performance Battery[19]

MMSE, Mini-Mental State Examination[18]

Table 2 shows the associations between the number of perceived environmental mobility barriers and type of mobility modification. The most common type of mobility modification was doing the task more slowly, while only fifteen participants reported that they had given up walking a 2 km distance. The number of perceived environmental barriers outdoors was associated with performing the task more slowly, and resting during performance of the task. The associations between the number of perceived environmental barriers and the use of assistive devices and reduction in the frequency of performing the task were borderline significant. No association was found between the number of perceived environmental barriers and having given up doing the task.

**Table 2:** Association between number of perceived environmental barriers outdoors and type of mobility modification among community-dwelling older people (n = 848).

		No. of perceived environment barriers			
		0	1	≥2	
Mobility modification	n	(n = 275)% (n)	(n = 177)% (n)	(n = 396)% (n)	P-value
Given up doing the task	15	4.3 (9)	3.9 (4)	1.1 (2)	.159
Doing slowly	134	17.3 (35)	28.7 (29)	39.3 (70)	**< .001**
Resting in the middle of the performance	55	7.4 (15)	9.0 (9)	17.4 (31)	**.007**
Using assistive device	46	6.4 (13)	8.9 (9)	13.5 (24)	.064
Reducing frequency	86	13.9 (28)	16.8 (17)	23.0 (41)	.063

P-value: Chi square test

Those reporting one environmental barrier had 1.7 times, and those reporting multiple environmental barriers had 2.4 times, the odds for mobility modification compared to those reporting no environmental barriers (age- and sex-adjusted). After adjusting for physical performance, several chronic conditions, cognitive functioning, and sociodemographic indicators, the association between mobility modifications and reporting one environmental barrier was at the borderline of statistical significance; however, for multiple environmental barriers the association remained statistically significant. The odds for manifest mobility difficulty were more than five times higher among those reporting multiple environmental barriers (Table 3).

**Table 3:** Multinomial logistic regression analyses on baseline associations between perceived environmental barriers outdoors and mobility modifications and mobility difficulty among community-dwelling older people (n = 848).

	Model 1				Model 2			
No. of Perceived Environmental Barriers	Mobility modification		Mobility difficulty		Mobility modification		Mobility difficulty	
	OR	95% CI	OR	95% CI	OR	95% CI	OR	95% CI
0	1.00		1.00		1.00		1.00	
1	**1.65**	**1.01–2.68**	**2.58**	**1.59–4.17**	1.55	0.93–2.60	**2.12**	**1.22–3.70**
≥2	**2.25**	**1.48–3.43**	**4.88**	**3.23–7.37**	**2.08**	**1.33–3.25**	**3.46**	**2.14–5.60**

Model 1 adjusted for age and gender

Model 2 adjusted for age, gender, SPPB, cognitive functioning, living alone, years of education, diabetes and pulmonary, cardiac, circulatory and neurological disease.

Reference group = those without mobility modification or difficulty.

### Longitudinal findings

Of those without manifest mobility difficulty at baseline (n = 492), 31% (n = 153) developed manifest mobility difficulty during the 2-year follow-up period. Reporting multiple (≥2) environmental barriers predicted development of mobility difficulty. Adjusting for several covariates did not materially change the result, but adding baseline mobility modifications into the model attenuated the association between the number of perceived environmental barriers and incident mobility difficulty (Table 4).

**Table 4:** Number of perceived environmental barriers and mobility modification as predictors of incident mobility difficulty among community-dwelling older people without mobility difficulty at baseline (n = 492).

		Model1		Model 2		Model 3	
	Cumulative incidence	OR	95% CI	OR	95% CI	OR	95% CI
No. of Perceived Environmental Barriers							
0	26.3%	1.00		1.00		1.00	
1	35.1%	1.49	0.87–2.54	1.43	0.82–2.52	1.34	0.74–2.37
≥2	40.4%	**1.83**	**1.17–2.87**	**1.75**	**1.09–2.82**	1.55	0.95–2.53
Mobility modification		-		-		**2.49**	**1.59–3.88**

Model 1 adjusted for age and gender

Model 2 adjusted for age, gender, SPPB, cognitive functioning, living alone, years of education, diabetes, and pulmonary, cardiac, circulatory and neurological disease.

Model 3 adjusted for age, gender, SPPB, cognitive functioning, living alone, years of education, diabetes, and pulmonary, cardiac, circulatory and neurological disease, and mobility modification

## Discussion

This study showed that the perception of multiple environmental mobility barriers outdoors increased the risk for incident mobility difficulty and that mobility modification attenuated the association, which is a novel finding. We also found that the perception of environmental mobility barriers outdoors increased the odds for mobility modification among community-dwelling older people, even after taking into account individuals’ health situation.

Our findings are in line with those of previous studies showing that environmental barriers precede the development of walking difficulties [11, 12, 14] and that mobility modifications also increase the risk for mobility difficulty[4, 5, 20, 21]. The higher risk for incident mobility difficulty among persons reporting multiple environmental barriers was not explained by the presence of other risk factors for mobility decline, such as lower extremity performance, chronic conditions or sociodemographic factors. Possible explanation for the association between environmental barriers and development of mobility difficulty is that environmental barriers increase feelings of insecurity [22] and thus restrict out-of-home activities in older people. This may potentially lead to physical inactivity [23] and further decline in walking ability [24–26]. The finding that mobility modification attenuated the risk that environmental mobility barriers pose for manifest mobility difficulty, supported our hypothesis. The findings are also supported by the “Disablement process model” by Verbrugge and Jette (1994), according to which a gap between environmental demands and personal capability can be narrowed through mobility modifications [13], as people modify their behaviour in an effort to optimize their person-environment fit [9, 27].

As suggested earlier, mobility modification may not always be a response to physical impairment [20]. In the present study we used mobility modification in walking 2 km, which is an indicator of early mobility decline. We suggest that a person’s living environment has a significant role in the adoption of mobility modification. Outdoor environments pose different kinds of challenges to mobility than those presented by indoor environments. Challenging environments increase the attentional requirements of walking as compared to walking on a smooth surface [28], while irregular surfaces influence gait patterns, in particular inducing slower walking speed and shorter step length, among older people [29–31]. These changes may accumulate and lead to other mobility modifications [13], such as the need to rest, especially when walking long distances outdoors.

In daily life, environmental characteristics perceived as problematic may hinder the possibility to run daily errands independently [32], and so predispose people to modify their behaviour. For example, if a person has a long distance to travel to a grocery store and the route is hilly with no resting places, he/she may first start to experience tiredness when performing this task. Such perceived difficulty, or an increased sense of insecurity [22], may lead to doing it more slowly and stopping to rest, and finally to reduce the frequency or giving it up altogether. It is possible mobility modification patterns form a hierarchy, some being easier to adopt than others; for example, using an assistive device may be preferred to giving up the activity. In a study among older people with osteoarthritis it was found that people tend to compensate for their impairments, for example, by performing their habitual tasks at a slower pace, rather than restricting their frequency or giving them up[33]. This was also the case in our study, as only a few participants reported that they had stopped walking 2-km distances, while doing the same task more slowly was the most common form of mobility modification, as also reported by Weiss and colleagues [20]. Further studies should address whether specific environmental barriers contribute to different kinds of mobility modifications; for example, do long distances to the grocery store increase the risk for reduced frequency of the activity, or does it more likely lead to the use of assistive devices? Identifying environmental challenges and their potential consequences would help in guiding older people with mobility impairment on ways of overcoming such challenges, and so increase their possibilities for outdoor mobility and participation in the community.

The strengths of the study are the large population-based sample of older community-dwelling people and the availability of good quality data. We conducted both cross-sectional and longitudinal analyses on a topic that has not been widely studied and offer novel findings on the relationship between perceived environmental barriers and the compensatory strategies for impaired mobility that older people use in their daily lives. The study also has its limitations. First, it is possible that some people report difficulty already when they have changed their way of doing a task (e.g. do the task more slowly than before), while some understand difficulty as the inability to perform a task [34]. Thus it is possible that some people who were categorized as “having difficulty” would better have been categorized as “no difficulty but mobility modification”. The fact that this was not done may have led to underestimation of the associations. Second, we used perceived environmental barriers instead of objectively measured environmental features. However, perceptions give us knowledge of the environmental barriers that a person actually encounters when moving outdoors and on the routes habitually used. Thus, when focusing on mobility modifications, perceived environmental barriers may be a more valid indicator than objectively measured neighbourhood features. Nevertheless, precisely how objective features of the environment, such as neighbourhood infrastructure, walkability and access to green areas influence mobility modifications and the development of mobility difficulty, is an interesting target for future research.

To conclude, our findings suggest that for older people who successfully modify their performance, the influence of the environment on incident mobility difficulty can be diminished. Older people use mobility modification to alleviate environmental press on mobility.
